# Anxiety, depression, and quality of life in children with solid tumors: a single-center exploratory study of sociodemographic, clinical, school, and social functioning correlates

**DOI:** 10.3389/fpsyg.2026.1834749

**Published:** 2026-06-22

**Authors:** Şule Çalışkan Kamış, Burak Kamış, Metin Çil, Nihal Boz, Begül Yağcı

**Affiliations:** 1 Department of Pediatric Hematology and Oncology, Adana Faculty of Medicine, Adana City Training and Research Hospital, University of Health Sciences, Adana, Türkiye; 2 Department of Child and Adolescent Mental Health and Diseases, Adana Faculty of Medicine, Adana City Training and Research Hospital, University of Health Sciences, Adana, Türkiye

**Keywords:** anxiety, child development, depression, pediatric oncology, quality of life, solid tumors

## Abstract

**Background:**

Children with solid tumors experience prolonged treatment, repeated hospital visits, and psychosocial stressors. This exploratory single-center cross-sectional study evaluated anxiety symptoms, depressive symptoms, and health-related quality of life in children with solid tumors and examined their associations with selected sociodemographic, clinical, school functioning, and social functioning variables.

**Methods:**

Fifty children aged 8–18 years with solid tumors were evaluated at a tertiary pediatric hematology-oncology center in Türkiye between August 2024 and August 2025. Validated Turkish versions of the Children's Depression Inventory (CDI), Screen for Child Anxiety Related Emotional Disorders (SCARED), Revised Child Anxiety and Depression Scale–Child Version (RCADS-CV), and Pediatric Quality of Life Inventory (PedsQL) were administered by a child and adolescent psychiatry specialist. The primary outcome was the SCARED total score. Sociodemographic and clinical variables were recorded.

**Results:**

The median age was 13 years. Clinically significant depressive symptoms (CDI ≥ 19) were detected in 16% of patients, and high anxiety risk (SCARED ≥25) in 56%. Median RCADS-CV total anxiety and depression scores were 42.0 and 44.5, respectively. Girls had higher PedsQL emotional functioning scores than boys (*p* < 0.001), whereas school functioning did not differ significantly between age groups (*p* = 0.124). In exploratory analyses, lower maternal education was associated with a higher likelihood of clinically significant depressive symptoms (Spearman's rho = −0.383, *p* = 0.006).

**Conclusion:**

Children with solid tumors showed a substantial burden of anxiety risk and depressive symptoms. Standardized psychosocial assessment may help identify children requiring emotional support and guide individualized, family-centered interventions. Larger longitudinal multicenter studies are needed to validate these exploratory findings.

## Introduction

The coexistence of psychiatric disorders in children with chronic illnesses can profoundly influence their academic life, psychosocial adjustment, emotional development, and peer relationships. Studies have demonstrated that children with chronic illnesses frequently display emotional lability, irritability, shyness, low tolerance for frustration, anxiety, and depressive symptoms (Kazak et al., 2015; Keng et al., 2022; Yildirim et al., 2017). Among pediatric patients with solid tumors, these difficulties may be further intensified by prolonged treatment courses, repeated hospital visits, uncertainty regarding prognosis, and disruption of normal developmental routines (Keng et al., 2022; Yildirim et al., 2017). These outcomes highlight the complex interplay between biological vulnerability, family dynamics, and environmental stressors.

The present study was guided by family systems and stress-coping perspectives, which emphasize that a child's psychological adjustment during chronic illness is influenced not only by disease-related stressors but also by family functioning, parental coping, communication patterns, and available caregiving resources (Kazak et al., 2015; Kearney et al., 2015). In pediatric oncology, these family-related factors may shape how children understand their illness, tolerate treatment-related stress, and adapt emotionally during treatment and follow-up.

Children with solid tumors often undergo prolonged hospitalization, invasive diagnostic and therapeutic procedures, school interruption, reduced peer interaction, and treatment-related physical changes (Yildirim et al., 2017). In addition, caring for a child with cancer places substantial emotional, social, and physical burdens on parents and caregivers. These burdens may disrupt family functioning, intensify parental distress, and influence the child's emotional wellbeing through reciprocal processes described in the Family Systems framework (Keng et al., 2022; Yildirim et al., 2017).

From the child's perspective, frequent hospital visits and prolonged stays can lead to alienation from peers and hinder the development of stable friendships. Younger children, who have not yet developed abstract thinking, may associate their illness with death or punishment, fostering confusion and feelings of guilt or worthlessness that underlie depressive states (Yildirim et al., 2017). During the school years, children may feel different from their peers because of short stature, alopecia, scars, or other visible treatment-related changes. These differences may make them feel left out or treated differently by others. In adolescence, increased self-awareness and social comparison may further increase vulnerability to emotional distress, particularly when the illness interferes with peer interaction, school participation, or age-appropriate social development. These developmental and peer-related challenges have been described in pediatric psycho-oncology and school reintegration literature (Thompson et al., 2015; Christiansen et al., 2015).

Peer relationships and social participation are important components of developmental adjustment in children and adolescents with cancer. Being perceived as “different” or “sick” may contribute to emotional distress, reduced social participation, and impaired health-related quality of life. Therefore, assessment of school and social functioning is clinically relevant in addition to symptom-based screening for anxiety and depression (Christiansen et al., 2015).

Beyond symptom burden, these psychosocial challenges may affect everyday functioning, including emotional adjustment, school participation, and broader health-related quality of life. Despite these concerns, data specifically focusing on anxiety, depression, and quality of life in children with solid tumors remain limited, particularly in single-center cohorts from middle-income settings.

Accordingly, the primary aim of this exploratory study was to evaluate anxiety symptoms, depressive symptoms, and health-related quality of life in children with solid tumors. The secondary aim was to examine whether these outcomes were associated with selected sociodemographic, clinical, school functioning, and social functioning variables.

By examining the association between parental education, sociocultural context, and children's psychological outcomes, we sought to emphasize the importance of culturally informed interpretation of psychosocial findings in pediatric oncology.

Given the limited literature in this field, we designed the study as an exploratory assessment of psychosocial burden rather than a test of causal relationships. This approach may provide insight into the necessity and structure of psychiatric and psychosocial support for children with solid tumors, guiding interventions tailored to individual and familial needs.

## Materials and methods

### Study design and setting

This single-center cross-sectional study was conducted in the Department of Pediatric Hematology and Oncology at the University of Health Sciences, Adana Faculty of Medicine, Adana City Education and Research Hospital, between August 1, 2024, and August 1, 2025. A total of 50 pediatric patients aged 8–18 years with a confirmed diagnosis of a solid tumor were included.

### Participants

Eligible participants were children aged 8–18 years who were being followed in our clinic during the study period, were able to understand and respond to the Turkish-language assessment tools, and were not receiving active psychiatric treatment at the time of evaluation. Both central nervous system and non-central nervous system solid tumors were eligible for inclusion. Because of the clinical heterogeneity of the cohort and the use of different staging systems across tumor types, patients were not grouped according to disease stage.

To ensure valid self-reporting, children were required to be sufficiently literate in Turkish to understand the questionnaire items and the clinician's instructions. Patients with severe cognitive impairment, marked memory problems, altered consciousness, or any condition preventing reliable completion of the self-report measures were excluded.

### Data collection

Sociodemographic and clinical variables were collected from medical records and caregiver interviews, including age (years), sex (female/male), residence (rural/urban), diagnosis category, disease status (remission/relapse), treatment modality (surgery, chemotherapy, and/or radiotherapy), time since diagnosis (months), parental education level (illiterate, primary school, middle school, high school, or higher education), academic performance (poor, average, good, or very good), sleep pattern (regular/irregular), daily screen exposure (<1, 1–2, or >2 h), surgical history (yes/no), and relapse status (yes/no).

Patients were evaluated when they were clinically stable and able to participate reliably in the interview and self-report questionnaires. No formal restriction was applied according to treatment phase; therefore, children could be assessed during active treatment or follow-up, and treatment-related variables were recorded for descriptive and exploratory analyses.

All questionnaires were administered in Turkish by a Child and Adolescent Psychiatry specialist in a semi-structured manner. Self-report forms were completed directly by the participants, whereas children with reading difficulties received clinician assistance during administration.

### Psychometric instruments

In this study, four standardized and validated assessment tools were used to evaluate anxiety, depressive symptoms, and health-related quality of life among pediatric patients: the Children's Depression Inventory (CDI), the Screen for Child Anxiety Related Emotional Disorders (SCARED), the Revised Child Anxiety and Depression Scale–Child Version (RCADS-CV), and the Pediatric Quality of Life Inventory (PedsQL). All scales were administered in Turkish and completed by the child, with clinician assistance provided when necessary for reading difficulties.

1. **Children's Depression Inventory (CDI):** The CDI is a 27-item self-report instrument developed to assess depressive symptoms in children and adolescents aged 7–17 years. Each item is rated on a three-point scale, reflecting the severity of symptoms over the past 2 weeks. A score of 19 or higher was considered indicative of clinically significant depressive symptoms based on the Turkish validity and reliability study of the Children's Depression Inventory (Öy, 1991).
2. **Screen for Child Anxiety Related Emotional Disorders (SCARED):** The SCARED is a 41-item Likert-type self-report instrument designed to assess anxiety symptoms in children and adolescents based on DSM-IV criteria. The total score ranges from 0 to 82, with higher scores indicating greater severity of anxiety. This scale is used to screen for anxiety disorders and to guide further evaluation or clinical intervention when needed. The Turkish version of the scale was validated by Karaceylan, and a cut-off score of 25 was used to indicate high anxiety risk (Karaceylan, 2005).
3. **The Revised Child Anxiety and Depression Scales-Child Version (RCADS-CV)**: The RCADS-CV is a 47-item self-report questionnaire designed to assess symptoms of anxiety and depression in children and adolescents based on DSM-IV diagnostic criteria. It includes six subscales: Separation Anxiety Disorder (seven items), Social Phobia (nine items), Generalized Anxiety Disorder (six items), Panic Disorder (nine items), Obsessive-Compulsive Disorder (six items), and Major Depressive Disorder (10 items). Each item is rated on a four-point Likert scale ranging from 0 (“never”) to 3 (“always”), reflecting the frequency of symptoms. The scale yields both individual subscale scores and composite indices for total anxiety and total depression. The Turkish RCADS-CV has demonstrated satisfactory psychometric properties in a clinical sample of Turkish children (Gormez et al., 2017).
4. **Pediatric Quality of Life Inventory (PedsQL):** The Pediatric Quality of Life Inventory (PedsQL) is a standardized tool used to evaluate health-related quality of life in children and adolescents aged 2–18 years. The scale comprises 23 items covering four core dimensions: physical functioning, emotional functioning, social functioning, and school functioning. Each item is rated on a five-point Likert scale ranging from 0 (“never”) to 4 (“almost always”), reflecting the frequency of problems experienced over the past month. Raw scores were transformed to a 0–100 scale, with higher scores indicating better quality of life. In this study, we used the child self-report Generic Core Scales for ages 8–18 years. The Turkish child self-report versions for 8–12 and 13–18 years have demonstrated good reliability and validity (Memik et al., 2007).

#### Sample size consideration

The study included 50 participants. The sample size was determined according to the average annual number of pediatric solid tumor cases followed at our institution and the feasibility of completing standardized psychiatric assessments during the study period. A formal *a priori* power analysis was not conducted; therefore, the sample size was considered feasibility-based and exploratory.

#### Missing data handling

Missing data were handled by complete-case analysis. No imputation method was used. Patients with incomplete questionnaire data were excluded from the final analytic dataset. For each statistical analysis, only cases with valid data for the relevant variables were included.

### Statistical analysis

All statistical analyses were performed using the Statistical Package for the Social Sciences (SPSS) version 26.0 (IBM Corp., Armonk, NY, USA). Categorical variables were presented as frequencies and percentages. Continuous variables were expressed as mean ± standard deviation, median [interquartile range (IQR)], or median (range), as appropriate. Normality of continuous variables was assessed using the Shapiro–Wilk test.

Because most psychometric variables were not normally distributed, non-parametric methods were preferentially used in the primary analyses. For group comparisons, Student's *t*-test was used for normally distributed continuous variables, and the Mann–Whitney *U* test was applied for non-normally distributed variables. Kruskal–Wallis tests were used for comparisons across screen-time categories, and Mann–Whitney *U* tests were used for two-group comparisons such as sex, age group, and sleep pattern.

Correlations involving non-normally distributed or ordinal variables were examined using Spearman's rank correlation coefficient. Associations between ordinal parental education variables and dichotomous psychological outcomes, such as CDI ≥ 19, were examined using Spearman's rank correlation and categorical cross-tabulation analyses. When appropriate, chi-square tests were used to compare categorical distributions across parental education groups. When expected cell counts were small, categorical test results were interpreted with caution. A *p* value of < 0.05 was considered statistically significant for all analyses.

Continuous descriptive variables, including maternal and paternal age, were summarized as median (range). Psychometric scale scores were summarized as median (IQR) when non-normally distributed.

Because item-level responses for the psychometric instruments were not available in the final analytic dataset, item-level Cronbach's alpha coefficients could not be recalculated for CDI, SCARED, RCADS-CV, or the original 23-item PedsQL. However, as a supplementary reliability analysis, Cronbach's alpha was calculated using the four PedsQL domain scores: physical functioning, emotional functioning, social functioning, and school functioning. This coefficient was interpreted as a domain-level internal consistency estimate and not as item-level reliability of the original PedsQL scale.

#### Ethical approval

This study was approved by the Adana City Training and Research Hospital Clinical Research Ethics Committee with the decision dated 25.07.2024 and numbered 71. Written informed consent was obtained from all participants' legal guardians. Child assent was obtained when developmentally appropriate. Children without parental consent or child assent were not enrolled in the study; therefore, lack of consent was not listed as an exclusion criterion.

## Results

Of the 50 patients included in the study, 23 were female and 27 were male. The median age was 13 years. Baseline sociodemographic and clinical characteristics are presented in Table 1. Thirteen patients had osteosarcoma, 10 had Ewing sarcoma, six had medulloblastoma, six had germ cell tumors, and the remaining patients had other solid tumors. Forty-one patients had undergone surgery, 39 were in remission, and 11 had experienced relapse. The median time since diagnosis was 14 months.

**Table 1:** Baseline sociodemographic and clinical characteristics of the patients.

Variable	Category	*n* (%)	Median (range)
Total number of patients		50 (100%)	
Sex	Female	23 (46%)	
	Male	27 (54%)	
Age, years			13 (8–18)
Age group	8–12 years	21 (42%)	
	13–18 years	29 (58%)	
Residence	Rural	12 (24%)	
	Urban	38 (76%)	
Maternal age, years			39 (28–56)
Paternal age, years			44 (35–63)
School level of the child	Primary school	14 (28%)	
	Middle school	18 (36%)	
	High school	18 (36%)	
Maternal education	Illiterate	7 (14%)	
	Primary school	23 (46%)	
	Middle school	10 (20%)	
	High school	5 (10%)	
	Higher education	5 (10%)	
Paternal education	Illiterate	4 (8%)	
	Primary school	20 (40%)	
	Middle school	6 (12%)	
	High school	13 (26%)	
	Higher education	7 (14%)	
Daily screen exposure	<1 h/day	11 (22%)	
	1–2 h/day	11 (22%)	
	>2 h/day	28 (56%)	
Sleep pattern	Regular	39 (78%)	
	Irregular	11 (22%)	
Academic performance	Very good (>85)	8 (16%)	
	Good (70–85)	17 (34%)	
	Average (60–70)	19 (38%)	
	Poor (<60)	6 (12%)	
Weekly exercise duration	0–1 h	9 (18%)	
	1–2 h	6 (12%)	
	>2 h	7 (14%)	
	No exercise	28 (56%)	
Diagnosis	Osteosarcoma	13 (26%)	
	Ewing sarcoma	10 (20%)	
	Medulloblastoma	6 (12%)	
	Germ cell tumor	6 (12%)	
	High-grade glioma	4 (8%)	
	Rhabdomyosarcoma	3 (6%)	
	Wilms tumor	2 (4%)	
	Neuroblastoma	2 (4%)	
	Retinoblastoma	1 (2%)	
	Optic glioma	1 (2%)	
	Ependymoma	1 (2%)	
	Fibrosarcoma	1 (2%)	
Surgery	Yes	41 (82%)	
	No	9 (18%)	
Disease status	Remission	39 (78%)	
	Relapse	11 (22%)	
Time since diagnosis, months			14 (2–48)

Values are presented as n (%) for categorical variables and median (range) for descriptive continuous variables.

**Figure 1:**
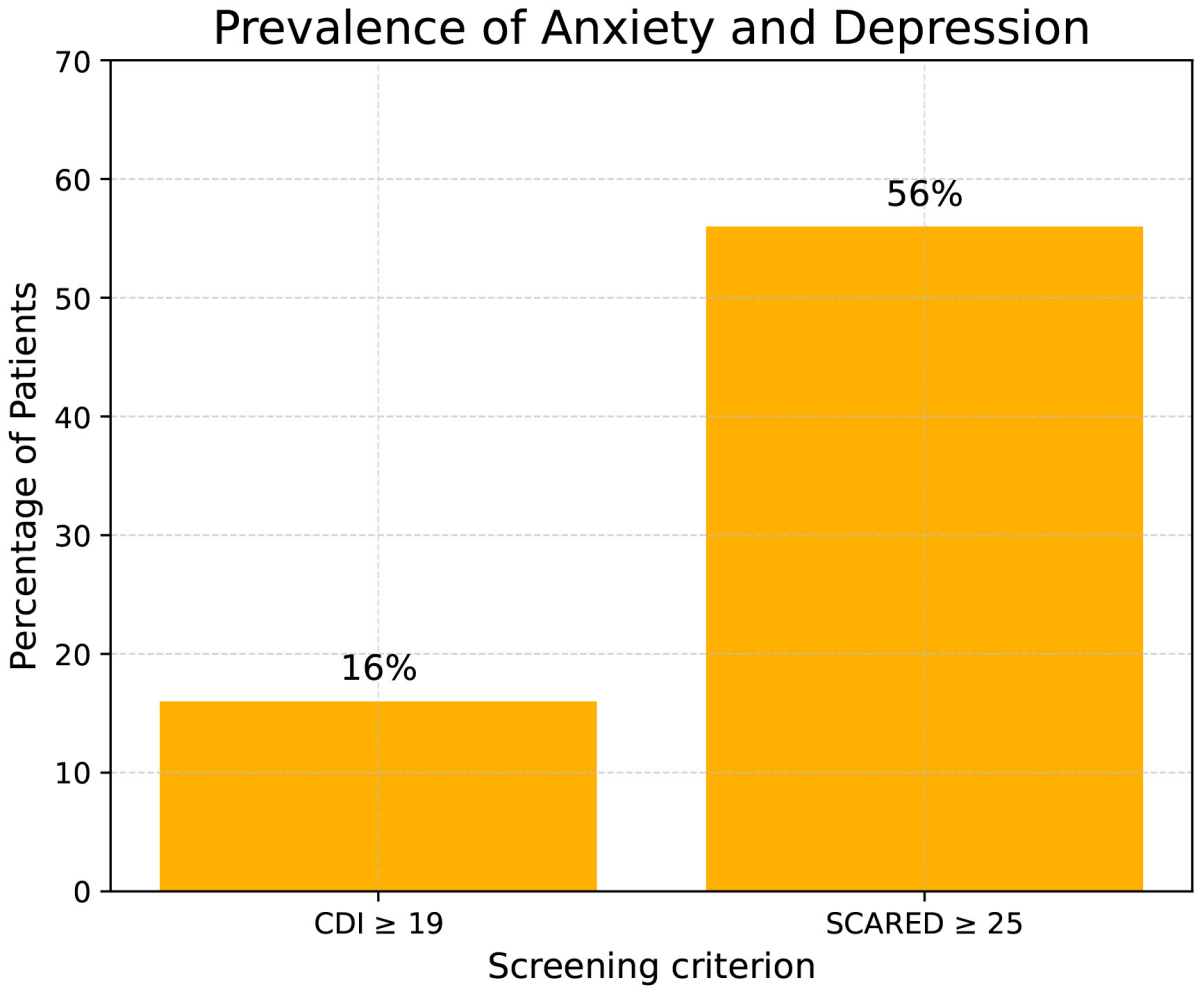
Prevalence of depressive symptoms and high anxiety risk in children with solid tumors. CDI, children's depression inventory; SCARED, screen for child anxiety related emotional disorders.

Eight patients (16%) scored ≥19 on the Children's Depression Inventory (CDI), indicating clinically significant depressive symptoms, and 28 patients (56%) scored ≥25 on the Screen for Child Anxiety Related Emotional Disorders (SCARED), indicating high anxiety risk (Table 2; Figure 1). The median RCADS-CV total anxiety and total depression scores were 42.0 (IQR, 38.75–53.0) and 44.5 (IQR, 39.0–56.0), respectively (Table 2).

**Table 2:** Psychological screening outcomes and scale scores.

Outcome domain	Instrument	Threshold or score	Result
Depressive symptoms	Children's depression inventory	CDI ≥ 19	8 (16%)
Anxiety risk	Screen for child anxiety related emotional disorders	SCARED ≥ 25	28 (56%)
Anxiety symptom burden	Revised child anxiety and depression scale-child version	Total anxiety score	42.0 (38.75–53.0)
Depression symptom burden	Revised child anxiety and depression scale-child version	Total depression score	44.5 (39.0–56.0)

CDI, children's depression inventory; SCARED, screen for child anxiety related emotional disorders; RCADS-CV, revised child anxiety and depression scale–child version; IQR, interquartile range.

In the supplementary reliability analysis, the four PedsQL domain scores demonstrated acceptable domain-level internal consistency, with Cronbach's alpha = 0.764 and Cronbach's alpha based on standardized items = 0.810 among 50 valid cases. This finding should be interpreted as a domain-score-based reliability estimate rather than item-level internal consistency of the original 23-item PedsQL.

PedsQL emotional functioning scores differed significantly by gender. Girls had higher emotional functioning scores than boys (mean rank 34.59 vs. 17.76; Mann–Whitney *U* = 101.5, *Z* = −4.082, *p* < 0.001). No significant differences were found between girls and boys in RCADS-CV total anxiety scores (Mann–Whitney *U* = 272.0, *p* = 0.453) or total depression scores (Mann–Whitney *U* = 250.5, *p* = 0.242).

No significant difference was found between the 8–12 and 13–18 age groups in PedsQL school functioning scores (mean rank 29.19 vs. 22.83; Mann-Whitney *U* = 227.0, *Z* = −1.539, *p* = 0.124). Similarly, no significant difference was found between the 8–12 and 13–18 age groups in RCADS-CV total anxiety scores (Mann–Whitney *U* = 270.5, *p* = 0.503) or total depression scores (Mann–Whitney *U* = 288.5, *p* = 0.753). The main group comparisons for psychometric outcomes are summarized in Table 3.

**Table 3:** Main group comparisons for psychometric outcomes.

Comparison	Outcome	Groups	Statistical result	*p* value
Sex	RCADS-CV total anxiety	Female vs. male	Mann-Whitney *U* = 272.0	0.453
Sex	RCADS-CV total depression	Female vs. male	Mann-Whitney *U* = 250.5	0.242
Sex	PedsQL emotional functioning	Female mean rank 34.59 vs. male mean rank 17.76	Mann-Whitney *U* = 101.5; *Z* = −4.082	< 0.001
Age group	RCADS-CV total anxiety	8–12 vs. 13–18 years	Mann-Whitney *U* = 270.5	0.503
Age group	RCADS-CV total depression	8–12 vs. 13–18 years	Mann-Whitney *U* = 288.5	0.753
Age group	PedsQL school functioning	8–12 years mean rank 29.19 vs. 13–18 years mean rank 22.83	Mann-Whitney *U* = 227.0; *Z* = −1.539	0.124

Mann-Whitney U tests were used for two-group comparisons. RCADS-CV, Revised Child Anxiety and Depression Scale-Child Version; PedsQL, Pediatric Quality of Life Inventory.

Exploratory correlation analysis using the recoded maternal education variable demonstrated a significant negative association between maternal education level and clinically significant depressive symptoms (CDI ≥ 19; Spearman's rho = −0.383, *p* = 0.006). In exploratory categorical analyses, the proportion of children with CDI ≥ 19 differed across maternal education groups (Pearson χ^2^ = 11.139, df = 4, *p* = 0.025; linear-by-linear association *p* = 0.013). Because most expected cell counts were below 5, this finding was interpreted cautiously.

The exploratory analyses of maternal education and clinically significant depressive symptoms are summarized in Table 4.

**Table 4:** Exploratory analyses of maternal education and clinically significant depressive symptoms.

Analysis	Comparison	Test statistic	*p* value
Spearman correlation	Maternal education (recoded) vs. CDI ≥ 19	rho = −0.383	0.006
Pearson chi-square test	Maternal education group vs. CDI ≥ 19	chi-square = 11.139; df = 4	0.025
Linear-by-linear association	Maternal education group vs. CDI ≥ 19	chi-square = 6.152; df = 1	0.013

CDI, children's depression inventory; df, degrees of freedom; rho, Spearman's rank correlation coefficient. Seven cells (70.0%) had expected counts below 5 in the chi-square analysis.

### Effect of screen time on psychometric outcomes

Exploratory analyses were performed to compare psychometric scores across daily screen-time categories (< 1, 1–2, and >2 h per day). Although some between-group differences were observed in anxiety and quality-of-life scores, these findings were not emphasized because of limited statistical robustness and the overall exploratory design of the study. Therefore, screen-time-related results should be interpreted cautiously and were not considered among the main findings of the present study. The exploratory comparison of psychometric scores across screen-time categories is presented in Table 5.

**Table 5:** Exploratory comparison of psychometric scores across screen-time categories.

Variable	<1 h median (IQR)	1–2 h median (IQR)	>2 h median (IQR)	Statistical result	*p* value
SCARED total	22 (18–27)	25 (20–30)	31 (25–36)	Kruskal-Wallis *H* = 7.14	0.028
CDI total	13 (10–17)	15 (11–18)	17 (12–19)	Kruskal-Wallis *H* = 2.46	0.292
PedsQL total	75 (69–81)	69 (64–75)	63 (57–70)	Kruskal-Wallis *H* = 6.52	0.038

Values are presented as median [interquartile range (IQR)]. h, hours/day; CDI, children's depression inventory; SCARED, screen for child anxiety related emotional disorders; PedsQL, pediatric quality of life inventory.

### Effect of sleep pattern on psychometric outcomes

The Shapiro–Wilk test indicated non-normal distributions for psychometric variables (*p* < 0.05). Therefore, Mann–Whitney *U* tests were performed to compare scores between children with regular and irregular sleep patterns. No statistically significant differences were observed in SCARED total scores (*U* = 191.5, *p* = 0.590), CDI scores (*U* = 173.0, *p* = 0.329), or PedsQL subscale scores, including physical health (*U* = 199.5, *p* = 0.725), emotional functioning (*U* = 207.5, *p* = 0.869), social functioning (*U* = 188.0, *p* = 0.532), and school functioning (*U* = 202.5, *p* = 0.776). These findings suggest that sleep pattern regularity was not significantly associated with anxiety, depressive symptoms, or quality-of-life outcomes in this cohort.

## Discussion

The present study found that children with solid tumors had a substantial psychosocial symptom burden, with 16% meeting the CDI screening threshold for clinically significant depressive symptoms and 56% meeting the SCARED threshold for high anxiety risk. RCADS-CV total anxiety and depression scores also indicated measurable emotional symptom burden. In addition, girls had higher PedsQL emotional functioning scores than boys, no significant age-group difference was found in school functioning, and lower maternal education was associated with a higher likelihood of CDI scores above the clinical threshold in exploratory analyses.

There is limited published evidence specifically addressing anxiety, depressive symptoms, and health-related quality of life in children with solid tumors. Previous pediatric oncology studies and recent reviews have shown that emotional distress may occur during treatment and follow-up and may be influenced by diagnosis, treatment phase, family functioning, caregiver-related factors, quality-of-life domains, and school or peer participation (Yildirim et al., 2017; Yardeni et al., 2021; Bakker et al., 2023; Bakula et al., 2020; Simard et al., 2024; Lietaviete and Martinsone, 2024; Inhestern et al., 2024). In this context, the present findings provide single-center exploratory data and support the need to evaluate emotional symptoms together with quality-of-life and family-related variables in children with solid tumors.

The observed rates of depressive symptoms and high anxiety risk suggest that emotional distress should not be regarded as a secondary or incidental issue in children with solid tumors. Recent pediatric psycho-oncology standards and systematic reviews support the integration of psychosocial assessment, school re-entry support, social participation, treatment adherence monitoring, family-centered interventions, and digital psychosocial support into pediatric oncology care (Kazak et al., 2015; Thompson et al., 2015; Christiansen et al., 2015; Simard et al., 2024; Pai and McGrady, 2015; Arpaci and Altay, 2024; Chandeying and Thongseiratch, 2021). Children who screen positive for depressive symptoms or high anxiety risk may benefit from structured child and adolescent psychiatric evaluation, psychoeducation, family counseling, and individualized supportive interventions.

Lower maternal education was associated with a higher likelihood of clinically significant depressive symptoms in exploratory analyses. This finding may reflect broader differences in caregiving resources, health literacy, illness-related communication, emotional burden, parental distress, or access to supportive resources within the family environment (Bakula et al., 2020; Simard et al., 2024; Lietaviete and Martinsone, 2024; Xin and Yu, 2024; Wu et al., 2022). However, parental understanding of the disease, health literacy, caregiving burden, and family communication were not directly assessed; therefore, maternal education should be interpreted as an exploratory marker of family-related context rather than as an isolated causal factor.

Girls had higher PedsQL emotional functioning scores than boys in the present cohort. This finding was unexpected and suggests that psychosocial responses in pediatric oncology may not follow a uniform gender pattern. Possible explanations include differences in coping strategies, emotional expression, perceived support, family responses, or interpretation of questionnaire items. Although previous studies suggest that age- and sex-related factors may influence health-related quality of life in children (Wang et al., 2022), the exploratory design and small sample size of the present study require cautious interpretation. This finding should therefore be evaluated in larger pediatric oncology cohorts.

No significant difference was found between the 8–12 and 13–18 age groups in PedsQL school functioning scores. Although school disruption is an important concern in pediatric oncology, our data did not demonstrate a statistically significant age-group difference in this domain. This may reflect heterogeneity in diagnosis, treatment phase, educational support, and school reintegration experiences. Regardless of statistical significance, school participation remains clinically important and should be considered in psychosocial care planning.

Exploratory analyses of screen-time categories suggested possible differences in anxiety and quality-of-life scores; however, these findings were not considered among the main findings because of the exploratory design and limited statistical robustness. Similarly, sleep pattern was not significantly associated with anxiety, depressive symptoms, or PedsQL domains in this cohort. Future longitudinal studies are needed to clarify whether daily routines such as screen exposure, sleep regularity, physical activity, and school participation meaningfully influence emotional and quality-of-life outcomes in children with solid tumors.

### Cultural context and interpretation

The association between lower maternal education and clinically significant depressive symptoms may also be interpreted within a broader sociocultural and family-centered context. In the Turkish sociocultural setting, mothers often assume a central role in daily caregiving, educational monitoring, and communication with healthcare professionals. Therefore, maternal education may be related not only to formal schooling but also to health literacy, access to support resources, illness-related communication, and the structure of emotional support during treatment (Yildirim et al., 2017; Karayagiz et al., 2020; Omer, 2024; Kagitcibasi, 2017). However, parental health literacy, caregiving burden, and family communication were not directly measured in this study; therefore, this interpretation remains exploratory.

### Limitations

This study has several limitations. First, the sample size was small and participants were recruited from a single tertiary center, which may limit generalizability. Second, the cohort was clinically heterogeneous, and diagnosis-specific, stage-based, treatment-specific, and multivariable analyses could not be performed. Third, the cross-sectional design prevents causal interpretation. Fourth, the study relied on child self-report measures, which may be affected by age, literacy, and response bias. Finally, parental health literacy, caregiving burden, family communication, and treatment adherence were not directly assessed. Therefore, all associations should be interpreted as exploratory.

Another limitation is that item-level responses for the psychometric instruments were not available in the final analytic dataset. Therefore, item-level Cronbach's alpha coefficients could not be recalculated for CDI, SCARED, RCADS-CV, or the original 23-item PedsQL. The reliability result reported for PedsQL should be interpreted only as a supplementary domain-level estimate based on the four PedsQL domain scores.

### Future directions

Future studies should validate these findings in larger, multicenter, and longitudinal cohorts. Further research should include both psychosocial and clinical variables, such as diagnosis group, treatment modality, relapse status, time since diagnosis, parental health literacy, caregiving burden, family communication, and school reintegration. Culturally adapted psychosocial interventions, including parental counseling, psychoeducation, stress management, resilience-based training, and digital support models, should also be evaluated to determine their effects on anxiety, depressive symptoms, and health-related quality of life in children with solid tumors (Simard et al., 2024; Arpaci and Altay, 2024; Chandeying and Thongseiratch, 2021; Ogez et al., 2019; Coughtrey et al., 2018; Melesse et al., 2022).

## Conclusion

In conclusion, children with solid tumors in this single-center cohort showed a substantial burden of anxiety risk and depressive symptoms. Standardized psychosocial assessment may help identify children who require emotional support and guide individualized, family-centered interventions. Future longitudinal multicenter studies are needed to clarify the clinical, sociodemographic, family-related, school-related, and social determinants of psychosocial outcomes in this population and to evaluate the effectiveness of targeted psychosocial interventions.

## Data Availability

The raw data supporting the conclusions of this article will be made available by the authors, without undue reservation.
